# Influence of epigallocatechin-3-gallate in promoting proliferation and osteogenic differentiation of human periodontal ligament cells

**DOI:** 10.1186/s12903-019-0768-7

**Published:** 2019-05-02

**Authors:** Jie Liu, Yi Lu, Jin Liu, Changxiong Jin, Yuchen Meng, Dandan Pei

**Affiliations:** 1 Key Laboratory of Shaanxi Province for Craniofacial Precision Medicine Research, College of Stomatology, Xi’an Jiaotong University, Xi’an, China; 2 Department of Prosthodontics, College of Stomatology, Xi’an Jiaotong University, 98 Xiwu Road, Xi’an, 710004 Shaanxi China; 3 Department of Periodontics, College of Stomatology, Xi’an Jiaotong University, Xi’an, China

**Keywords:** Epigallocatechin-3-gallate, Human periodontal ligament cells, Proliferation, Osteogenic differentiation

## Abstract

**Background:**

Epigallocatechin-3-gallate (EGCG) was recently proposed to have the potential to regulate bone metabolism, however, its influence on osteogenesis remains controversial. The present study aimed to investigate the effects of EGCG on the proliferation and osteogenesis of human periodontal ligament cells (hPDLCs).

**Methods:**

Cells were cultured in osteogenic medium and treated with EGCG at various concentrations. Cell proliferation was analyzed using a CCK-8 assay and acridine orange (AO)/ethidium bromide (EB) staining. Flow cytometry was used to measure the intracellular reactive oxygen species (ROS) potential of hPDLCs. The expression levels of osteogenic marker genes and proteins in hPDLCs, including type I collagen (COL1), runt-related transcription factor 2 (RUNX2), osteopontin (OPN), and osterix (OSX), were determined by quantitative real-time polymerase chain reaction (qRT-PCR) and western blot analysis. In addition, alkaline phosphatase (ALP) activity was monitored both quantitatively and qualitatively. Extracellular matrix mineralization was further analyzed by alizarin red S staining.

**Results:**

The results showed that EGCG concentrations from 6 to 10 μM increased the ROS level and inhibited the cell proliferation of hPDLCs. EGCG concentrations from 2 to 8 μM effectively increased extracellular matrix mineralization, in which 4 and 6 μM EGCG generated the most mineralizing nodules. The ALP activity and the mRNA and protein expression levels of the tested osteogenic markers were most strongly up-regulated by treatment with 4 and 6 μM EGCG.

**Conclusions:**

The present study demonstrated that EGCG might promote the osteogenesis of hPDLCs in a dose-dependent manner, with concentrations of 4 and 6 μM EGCG showing the strongest osteogenic enhancement without cytotoxicity, indicating a promising role for EGCG in periodontal regeneration in patients with deficient alveolar bone in the future.

## Background

The periodontium, consisting of the alveolar bone, gingiva, periodontal ligament, and cementum, serves as a potent support for tooth stability, nutrition and resistance to stress during mastication movement. Based on an epidemiological investigation, the major cause of bone loss in the periodontium has been considered to be periodontitis, which is highly prevalent and affects up to 538 million people worldwide, and this number is likely to increase as many populations continue growing [1]. The destruction of periodontal bone tissue due to severe periodontitis may result in gingival recession, uncomfortable tooth mobility, and eventual tooth loss [2]. The restoration of damaged periodontal tissue, especially the alveolar bone, is the ultimate purpose of periodontal therapy. In routine clinical settings, antimicrobial, non-surgical, and surgical approaches are currently being used, either separately or in combination, to prevent the progression of periodontitis. Although these treatments are effective for controlling periodontal disease, they are not competent for predictable regeneration. Hence, seeking an effective method for periodontal regeneration is of paramount importance for both dentists and patients.

Epigallocatechin-3-gallate (EGCG), which is the most abundant and functional catechin polyphenol in green tea, has gained great attention in the past decades due to its multiple health benefits [3]. As a natural compound, EGCG has been reported to have antimicrobial, antioxidant, antitumor, antiviral, and antimutagenic effects in systemic diseases [3, 4]. Furthermore, several studies have indicated that local drug therapy using green tea could reduce gingival inflammation and improve periodontal parameters, such as probing depth, bleeding on probing and clinical attachment loss [5–7]. The biological effects of EGCG with regard to improving periodontal health may be achieved through the inhibition of the adherence of *Porphyromonas gingivalis* and the disruption of the initial step of biofilm formation [8, 9]. These findings indicate that EGCG might be a promising alternative for treating patients with periodontitis.

In addition to the abovementioned biological effects of EGCG, recently, EGCG was reported to have the ability to regulate bone metabolism [10–12]. Bone remodeling is regulated by the coupled actions of osteoblasts and osteoclasts [13, 14]. The primary pathogenesis of osteopenic diseases, including periodontitis, is the imbalance between osteoblastic activity and osteoclastic activity. Previous studies demonstrated that EGCG could inhibit pro-inflammatory cytokine expression, including tumor necrosis factor-α (TNF-α) and interleukin-6 (IL-6), which are responsible for the reduction on osteoclastic activity [13, 15], or even induce the apoptosis of osteoclasts to inhibit bone loss [16, 17]. Aside from these studies, which have focused on the suppression of osteoclasts, the effects of EGCG on osteogenesis have not been clearly elucidated thus far. Epidemiological investigations have suggested a close association between bone mineral density and consumption of green tea, which is the primary main source of EGCG for humans with osteoporosis [18]. Vali et al. [19] found that EGCG increased alkaline phosphatase (ALP) activity and the formation of mineralized bone nodules in SaOS-2 human osteoblast-like cells. Thus, it was hypothesized that EGCG possessed the potential to promote osteogenesis. However, conflicting results reported that EGCG appeared to inhibit the osteogenic differentiation of MC3T3-E1 cells [20], and another study reported that ALP activity and calcium content were repressed by EGCG in an ectopic bone formation model [21]. Thus, further studies are required to provide evidence for this controversial topic.

Human periodontal ligament cells (hPDLCs), which are constitutive of several cell types and possess high self-renewal and pluripotency potential, may serve as seed cells in periodontal bone regeneration [22, 23]. Studies that used hPDLCs for bone regeneration indicated that hPDLCs could differentiate into osteoblast-like cells and construct alveolar bone-like tissues [23, 24]. In the present study, we aimed to investigate the effects of different concentrations of EGCG on the cell proliferation and osteogenesis of hPDLCs to clarify the role played by EGCG in osteogenesis and its potential applications for periodontal bone regeneration.

## Materials and methods

### Cell isolation and culture

The present study was approved by the Ethics Committee of College of Medicine & Hospital of Stomatology, Xi’an Jiaotong University (approval number xjkqll[2016]NO.048). Primary periodontal ligaments were obtained from human premolars that were extracted for orthodontic reasons. Briefly, the periodontal ligament tissues attached to the mid-third of the root surfaces were scraped off with a sharp surgical scalpel, followed by treatment with 1 mg/mL type I collagenase (Gibco, Los Angeles, CA, USA) for 30 min. Then, the tissues were cultured in dishes with growth medium at 37 °C, in air with 95% humidity plus 5% CO_2_, and the medium was changed every 3 d. The growth medium consisted of 89% α-minimum essential medium (α-MEM; Invitrogen, USA), 10% (*v*/v) fetal bovine serum (FBS; Gibco), and 1% antibiotic (100 U/ml streptomycin and 100 U/ml penicillin, both from Gibco). When the cells reached 70–80% confluence, they were digested with 0.25% trypsin (Gibco) for further passaging. Passages 3 to 6 were used in the present study.

### EGCG treatment

EGCG was purchased from Sigma-Aldrich (St. Louis, MO, USA) and dissolved in distilled water to prepare a 10 mM stock solution according to the manufacturer’s instructions. Due to light and temperature sensitivity, the stock solution of EGCG was protected from light and maintained at − 20 °C until use. Prior to application to cells, the EGCG stock solution was diluted with prewarmed growth medium at 37 °C. For subsequent experiments, hPDLCs were cultured with EGCG at concentrations of 0 (the control group), 2, 4, 6, 8 and 10 μM in either growth medium or osteogenic differentiation medium (ODM) for the specified times prior to further experiments. The ODM consisted of the growth medium, as described above, with additional supplementation of 50 μM L-ascorbic acid, 0.1 μΜ dexamethasone and 10 mM β-glycerophosphate (all purchased from Sigma-Aldrich). The medium was renewed every 3 d.

### Cell proliferation

The Cell Counting Kit-8, purchased from Dojindo (CCK-8; Kumamoto, Japan), was used to explore the role played by EGCG on the proliferation of hPDLCs, according to the manufacturer’s instructions. Briefly, hPDLCs at a density of 2 × 10^3^ cells per well were seeded into clear-bottomed 96-well culture plates and cultured for 24 h. Subsequently, the growth medium was replaced by medium in which EGCG had been added at the various concentrations mentioned above. At time points of 24, 48 or 72 h, 10 μL of CCK-8 reagent was added to each tested well and incubated at 37 °C for 1 h, protected from light. The absorbance value was recorded by a Thermo Scientific microplate absorbance reader at 450 nm (Shanghai, China).

### Acridine orange/ethidium bromide (AO/EB) staining

To estimate the cell viability following EGCG treatment, hPDLCs were stained with a dual-fluorescence solution consisting of AO and EB (Sigma-Aldrich), according to the manufacturer’s instructions. Generally, AO penetrates living cells with intact membranes and then binds to DNA to generate green fluorescence, while EB only enters dead cells with damaged membranes to generate red fluorescence. The hPDLCs were seeded in 6-well plates at a density of 1 × 10^5^ cells per well and incubated in the growth medium for 24 h. After removing the growth medium, fresh medium containing the abovementioned concentrations of EGCG was added, and the cells were cultured for 24 h. Then, 300 μL of the dual-fluorescence staining solution, containing 0.1 g/L AO and 0.1 g/L EB at a proportion of 1:1, was added to each well and incubated for 10 min. Fluorescence was viewed through an Olympus FSX100 fluorescence microscope (Olympus Corporation, Tokyo, Japan).

### Quantification of reactive oxygen species (ROS)

ROS levels were detected using 2′, 7′-dichlorofluorescein diacetate (DCFH-DA; Beyotime, Shanghai, China) according to the manufacturer’s instructions. The cell culture and EGCG treatment protocols were the same as those described for the AO/EB staining assay. After culturing for 72 h, the hPDLCs were incubated with 200 μL growth medium supplemented with 10 μM DCFH-DA at 37 °C for 30 min. Then the cells were harvested and measured by flow cytometry (BD Biosciences, San Jose, CA, USA) with an excitation at 490 nm and an emission at 530 nm. The generation of intracellular ROS was then quantified.

### ALP activity

A Quantichrom Alkaline Phosphatase Assay Kit (Nanjing Jiancheng Bioengineering Institute, Nanjing, China) was used to evaluate the effects of EGCG on the intracellular ALP activity of hPDLCs. The cells were seeded into a 24-well plate at a density of 2 × 10^4^ cells per well and cultured in growth medium for 24 h. Then the medium was replaced with ODM containing EGCG at the concentrations mentioned above and cultured for 7 d. The cells were scraped off and solubilized in 200 μL lysis buffer containing 0.2% Triton-X 100 (Sigma-Aldrich) for 30 min at 37 °C. After that, the cell lysates were centrifuged to remove the cell impurities. ALP activity was then tested according to the manufacturer’s protocol. Then, 30 μL of the obtained supernatant and 100 μL of ALP substrate solution were transferred to a new clear-bottomed 96-well plate. After incubation for 15 min at 37 °C, 150 μL of coloration reagent was added to each well, and the samples were measured at a 520 nm wavelength with a microplate reader. The ALP activity was calculated against the total protein content, as determined by a Pierce™ BCA Assay Kit (Thermo Scientific) and was expressed as light units/mg protein. Besides, the BCIP/NBT Alkaline Phosphatase Staining Kit (Beyotime) was used according to the manufacturer’s instructions, on day 7 for qualitative observation.

### Alizarin red S assay

The alizarin red S assay was performed to assess extracellular matrix mineralization both qualitatively and quantitatively according to the manufacturer’s instructions. The cells were cultured as described for the ALP assay. After osteogenic incubation for 21 d, the cells were fixed with 4% paraformaldehyde for 30 min, stained with 40 mM alizarin red S (Sigma-Aldrich) at pH 4.2 for 30 min, and then photographed for the qualitative evaluation of mineralizing nodules. For quantification, 10% acetic acid was added to the sample plates to dissolve the stained mineral nodules at room temperature with gentle shaking. After 30 min, 10% ammonium hydroxide was used to neutralize the acetic acid. A microplate reader was employed to read the absorbance at 405 nm. The concentration of alizarin red S in each sample was determined by comparing the absorbance to a standard curve of known alizarin red S concentrations. The determination was based on the assumption that an alizarin red S - calcium complex was produced by 1 mol of alizarin red S binding to 2 mol of calcium [25]. Therefore, the results of extracellular mineralization, as determined through alizarin red S staining, were expressed as the calcium concentration ultimately.

### Quantitative real-time polymerase chain reaction (qRT-PCR)

QRT-PCR was used to detect the relative changes in the mRNA expression levels of runt-related transcription factor 2 (*RUNX2*), osteopontin (*OPN*), osterix (*OSX*) and type I collagen (*COL1*) which are recognized as markers of osteogenic differentiation. Cells were cultured in six-well plates with growth medium at a density of 1 × 10^5^ cells per well for 24 h. Then the culture medium was replaced with ODM containing EGCG at the previously mentioned concentrations and renewed every 3 d. After 7 d, the cells were lysed using TRIzol Reagent (Invitrogen) to extract the total mRNA and then converted to synthesize cDNA using a Primer Script® RT Reagent Kit (TaKaRa, Tokyo, Japan). The sequences of the primers used in the present study are shown in Table 1. Finally, the obtained cDNA was amplified with SYBR Green PCR Master Mix (TaKaRa) in an ABI 7500 Real-Time PCR system (Applied Biosystems, Singapore). The reaction conditions for qRT-PCR were set according to the manufacturer’s instructions. The relative mRNA expression levels were calculated using the 2^-△△Ct^ method, in which the mRNA expression levels of the selected osteogenic genes were normalized to the mRNA expression level of *GAPDH*. The results were calculated as fold changes.

**Table 1:** Primers for polymerase chain reaction

Gene name	Forward primer	Reverse primer
*COL1*	AGAGGAAGGAAAGCGAGGAG	GGACCAGCAACACCATCTG
*RUNX2*	GACGAGGCAAGAGTTTCACC	GGTTCCCGAGGTCCATCTAC
*OPN*	GCCGAGGTGATAGTGTGGTT	ACTCCTCGCTTTCCATGTGT
*OSX*	CCCACCTCAGGCTATGCTA	CACTGGGCAGACAGTCAGAA
*GAPDH*	GCACCGTCAAGGCTGAGAAC	TGGTGAAGACGCCAGTGGA

*COL1* type I collagen, *RUNX2* runt-related transcription factor 2, *OPN* osteopontin, *OSX* osterix, *GAPDH* glyceraldehyde-3-phosphate dehydrogenase

### Western blot

Western blot was used to analyze the protein expression levels of COL1, RUNX2, OPN and OSX. The hPDLCs were treated as described for the qRT-PCR assay. The cells were disrupted in pre-cooled RIPA buffer (Beyotime) after being treated with EGCG for 14 d. The protein content in the extracted cell lysates was determined using a Pierce™ BCA Assay Kit (Thermo Scientific) according to the manufacturer’s instructions. The obtained proteins (20 μg) were separated by 8~12% SDS-polyacrylamide gels. Then, the proteins were transferred onto polyvinylidene difluoride membranes (PVDF; Millipore, MA, USA) with a pore size of 0.45 μm, followed by blocking with 5% fat-free dry milk for 3 h. Subsequently, the membranes were incubated with individual primary antibodies at 4 °C overnight. The following antibodies were used: anti-COL1 (ab138492, Abcam, MA, USA), anti-RUNX2 (ab23981, Abcam), anti-OPN (ab91655, Abcam) and anti-OSX (ab22552, Abcam). The membranes were then incubated with the secondary antibody (ab205718, Abcam) conjugated with horseradish peroxidase for 1 h. The protein bands on the PVDF membranes were then stained using an enhanced chemiluminescence reagent. The stained bands were visualized using the Image Studio Lite software (Media Cybernetics Inc., Bethesda, MD, USA) and quantified by comparing the band intensities to that of GAPDH.

### Statistical analysis

All tests were performed three times which consisted of more than three independent experiments. The results were analyzed using SPSS 18.0 software (IBM, Chicago, IL, USA). For ROS assay, ALP activity assay, alizarin red S assay, qRT-PCR and western blot, one-way ANOVA followed by Tukey’s multiple post hoc tests was used to evaluate the differences among groups after normality (Shapiro–Wilk test) and homoscedasticity (modified Levene test) were assessed. Data were expressed as mean ± standard deviation. For the CCK-8 assay, two-way ANOVA followed by Tukey’s multiple tests was used. Data were expressed as median and interquartile range (IQR). Significant differences were set in advance at *p* < 0.05.

## Results

### Cell viability and proliferation

**Fig. 1:**
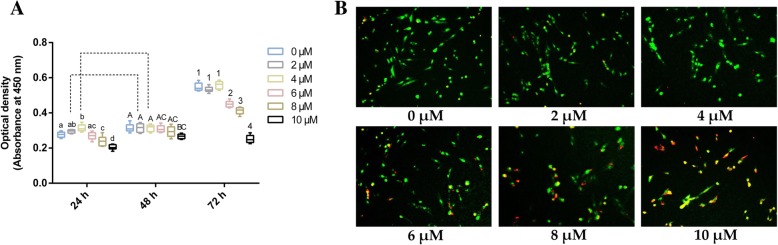
Effects of EGCG on cell proliferation of hPDLCs. (A**)** Cell proliferation was determined using CCK-8 assay after EGCG treatment for 24, 48 or 72 h (*n* = 5). Boxplots represent median and interquartile range (IQR). For the factor “concentration of EGCG” in each chart, groups at the same time-point labeled with different letters (lower case letters for 24 h, upper case letters for 48 h and numbers for 72 h) are significantly different from each other (*p* < 0.05) and those with the same letters exhibit no significant difference. For the factor of “culture time” in each chart, groups treated with the same concentration of EGCG that are connected with a horizontal bar are not significant; (B) Representative microscopic fluorescence images of AO/EB stained specimens after EGCG treatment for 72 h (*n* = 3). Green fluorescence indicates living cells, while red fluorescence indicates apoptotic cells. Bar, 100 μm

The effects of EGCG on cell proliferation, as determined by the CCK-8 assay, are shown in Fig. 1A. Two-way ANOVA revealed that the concentration of EGCG (*F* = 16.090, *p* < 0.05) and the culture time (*F* = 112.040, *p* < 0.05) both significantly influenced the proliferation of hPDLCs. The interaction between EGCG concentration and culture time was also significant (*F* = 3.827, *p* < 0.05). The groups treated with 0, 2 and 4 μM EGCG showed comparable levels of cell proliferation for all of the tested time points, 24, 48 and 72 h, except for the 4 μM EGCG group at 24 h, while concentrations of 8 and 10 μM EGCG had adverse effects on the proliferation of hPDLCs. No significant differences were found between the 6 μM EGCG group and the control group at 24 or 48 h. A decrease in proliferation was found in the 6 μM EGCG group after culturing for 72 h when compared to the control group.

The cell viability results, as detected by AO/EB staining, are shown in Fig. 1B. The cells in the groups treated with 0 to 6 μM EGCG primarily exhibited green fluorescence, indicating that these cells were in a healthy state with a fine form of nucleus. The number of apoptotic cells stained with red fluorescence increased in the groups treated with 8 and 10 μM EGCG. Cell apoptosis was more significant in the 10 μM EGCG group than that in the 8 μM EGCG group.

### Intracellular ROS expression

**Fig. 2:**
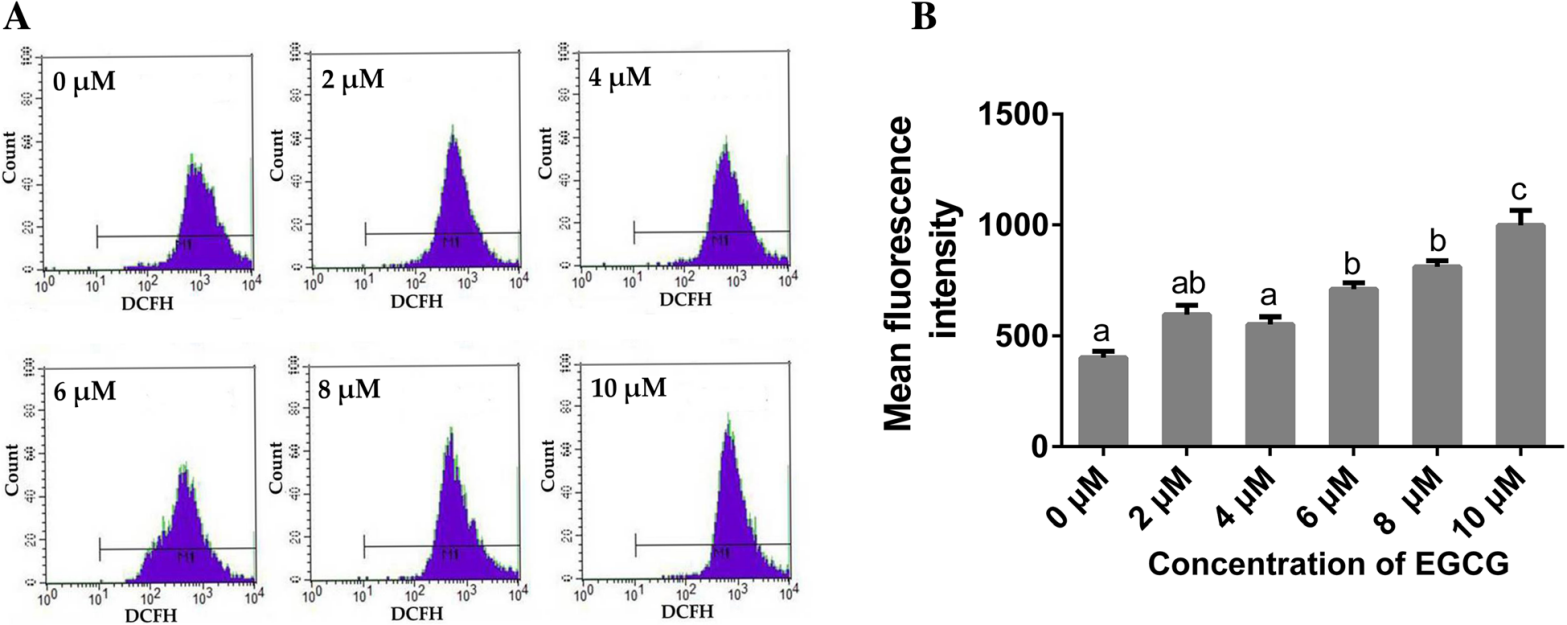
Effects of EGCG on the generation of intracellular ROS in hPDLCs. (A) Representative images of intracellular ROS levels determined by flow cytometry after the cells were treated with EGCG for 72 h (*n* = 5); (B) Quantitative analysis of intracellular ROS levels (*n* = 5). Data are presented as mean ± standard deviation. The bars with different lower case letters are significantly different from each other (*p* < 0.05), and those with the same letter exhibit no significant difference

Figure 2 presents the expression levels of intracellular ROS after EGCG treatment. ROS appeared to be induced by EGCG in a dose-dependent manner. No significant differences in the production of ROS were found among the groups treated with 0, 2 or 4 μM EGCG. Treatment with 6 to 10 μM EGCG induced more ROS when compared to the control group, while no significant differences were found between the 6 and 8 μM EGCG groups. The 10 μM EGCG group exhibited the highest level of ROS expression among all the groups.

### ALP activity and extracellular matrix mineralization

**Fig. 3:**
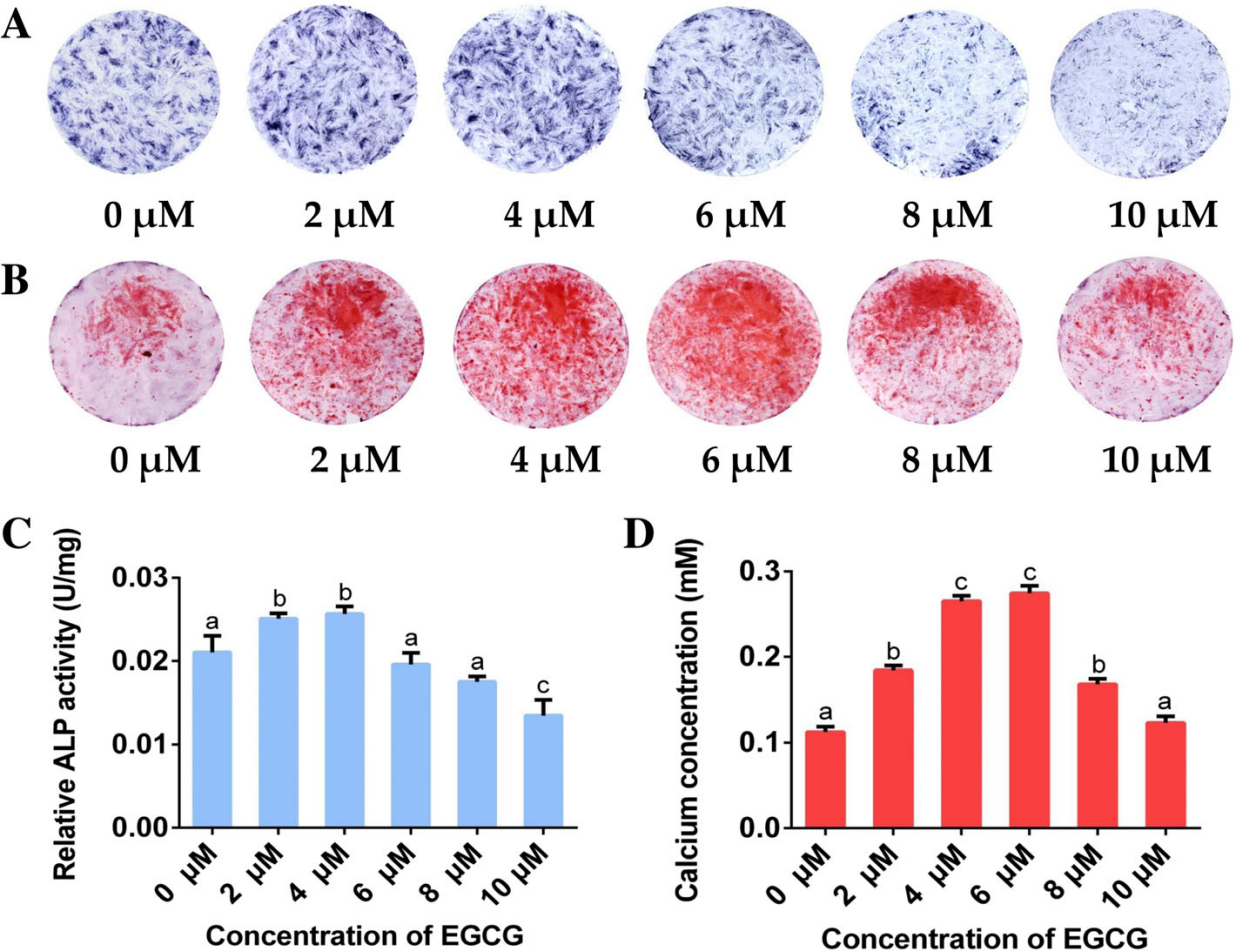
Effects of EGCG on ALP activity and extracellular matrix mineralization in hPDLCs. (A) Representative images of ALP enzyme staining after EGCG treatment for 7 d (*n* = 3); (B) Representative images of alizarin red S staining after EGCG treatment for 21 d (*n* = 3); (C) Quantitative analysis of ALP activity after EGCG treatment for 7 d (*n* = 5); (D) Quantitative analysis of alizarin red S staining after EGCG treatment for 21 d (*n* = 5). Data are presented as mean ± standard deviation. The bars with different lower case letters are significantly different from each other (*p* < 0.05), and those with the same letter exhibit no significant difference

Images of the hPDLCs after ALP staining are shown in Fig. 3A. The quantitative analysis of ALP activity is shown in Fig. 3C. It was revealed that ALP activities in the 2 and 4 μM EGCG groups were significantly higher than those in the other groups, while the 10 μM EGCG group showed remarkably lower ALP activity among all of the groups tested. In addition, no significant differences in ALP activity were found among groups treated with 0, 6 or 8 μM EGCG.

As shown in Fig. 3B, mineralizing nodules were found in all of the groups after cell culture for 21 d. Quantitative analysis of the calcium content in the mineralizing deposits is shown in Fig. 3D. Except for the 10 μM EGCG group, all of the groups exhibited a higher degree of mineralization than the control group. The 4 and 6 μM EGCG groups showed the highest calcium contents in the extracellular matrix among all of the groups, and no significant differences were found between these two groups.

### Gene expression

**Fig. 4:**
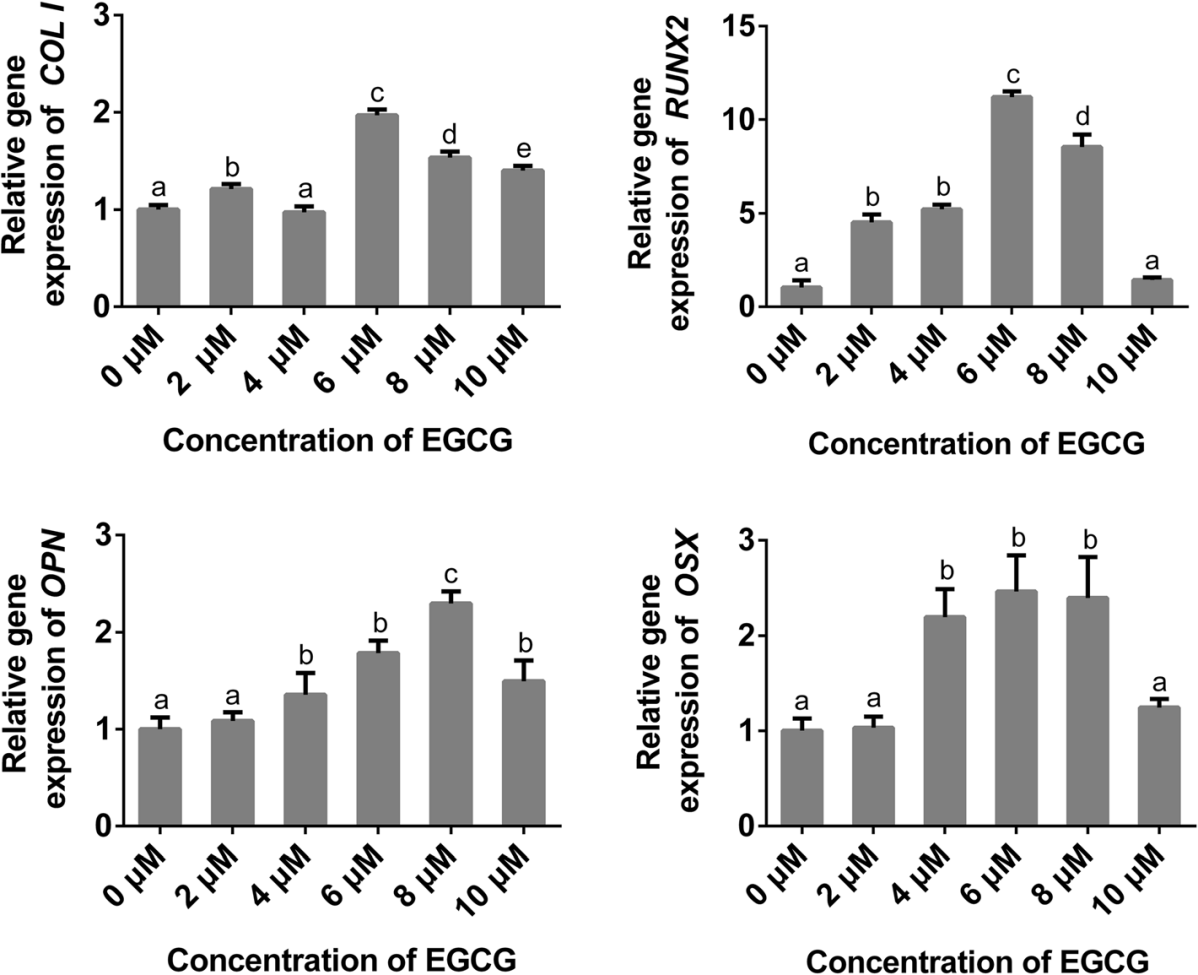
Relative expression levels of *COL1*, *RUNX2*, *OPN* and *OSX* measured by qRT-PCR after treatment with EGCG for 7 d. Semi-quantitative analysis of relative gene expression levels. Data are presented as mean ± standard deviation (*n* = 5). The bars with different lower case letters are significantly different from each other (*p* < 0.05), and those with the same letter exhibit no significant difference

EGCG treatment significantly increased the mRNA levels of *COL1, RUNX2, OPN* and *OSX* in most of the treatment groups, as shown in Fig. 4. The groups treated with 4 to 8 μM EGCG showed a remarkable increase in the expression levels of the osteogenic related genes tested in the present study. For *COL1, RUNX2* and *OSX*, the gene expression levels peaked in the 6 μM EGCG group. For *OPN*, the gene expression levels peaked in the 8 μM EGCG group.

### Protein expression

**Fig. 5:**
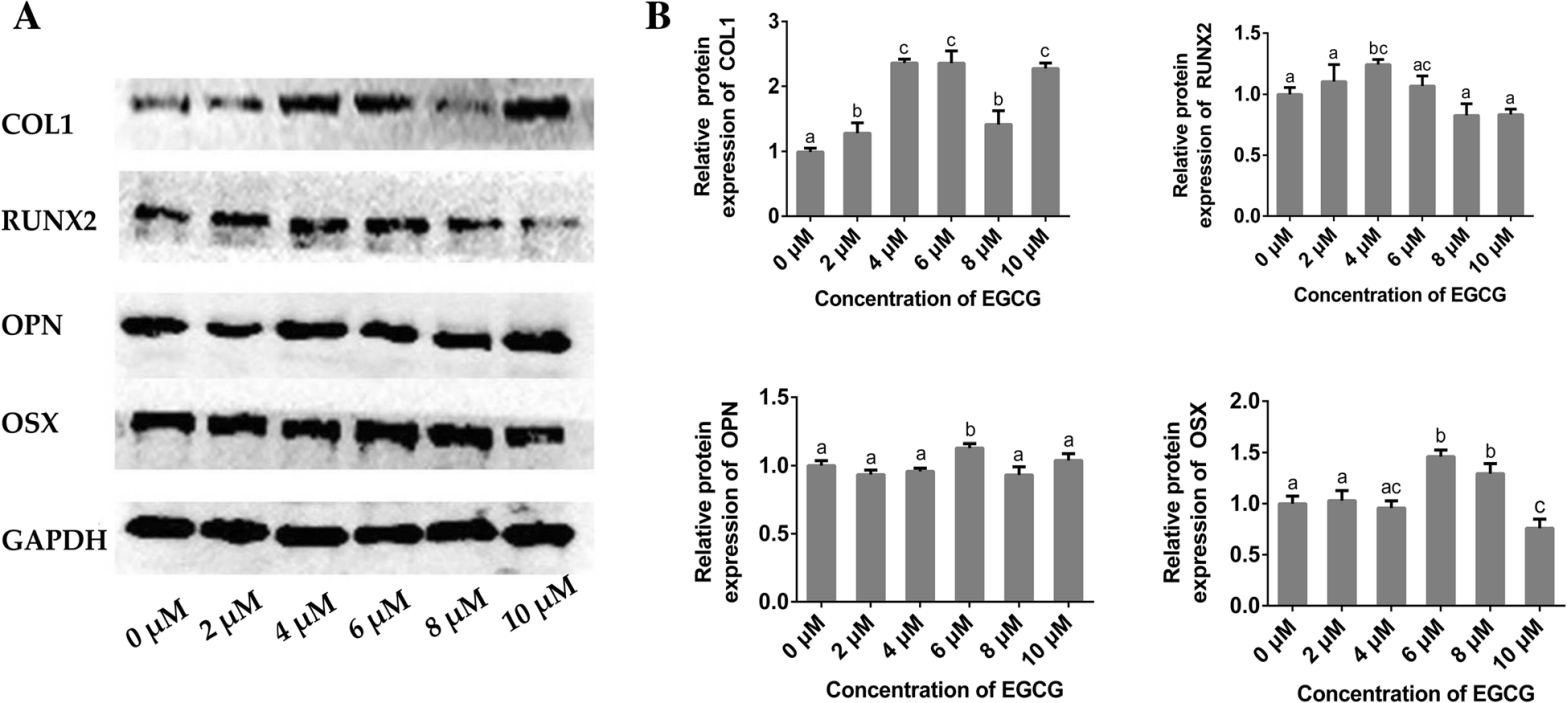
Relative expression levels of COL1, RUNX2, OPN and OSX measured by western blot after treatment with EGCG for 14 d. (A) Representative western blot scans of each protein after treatment with EGCG; (B) Semi-quantitative analysis of relative protein expression levels. Data are presented as mean ± standard deviation (*n* = 5). The bars with different lower case letters are significantly different from each other (*p* < 0.05), and those with the same letter exhibit no significant difference

The results of the protein expression analysis using western blot are shown in Fig. 5. Compared to the control group, the protein levels of COL1 were significantly increased in all of the EGCG groups. For RUNX2, only the 4 μM EGCG treatment up-regulated the protein levels significantly. Treatment with 2 and 4 μM EGCG had no effect on the expression levels of OPN and OSX. While treatment with 6 μM EGCG up-regulated the protein levels of OPN and OSX. The 10 μM EGCG treatment decreased the protein levels of OSX when compared to the control group.

## Discussion

Using bone augmentation procedures to promote periodontal bone regeneration is the dominant strategy for reestablishing both function and aesthetics in patients with deficient alveolar bone [26]. Currently, green tea is one of the most popular beverages consumed worldwide. As the most abundant catechin and a major bioactive component of green tea, EGCG has been reported to be associated with bone metabolism [13]. However, the specific effects of EGCG on osteogenic differentiation have been ambiguous. In the present study, the effects of EGCG on the proliferation and osteogenesis of hPDLCs were evaluated to clarify its potential role in periodontal bone regeneration.

Primary-cultured hPDLCs were used in the present study. Cell proliferation was evaluated by CCK-8 assay and AO/EB staining. The results suggested that supplementation with 4 μM EGCG significantly increased the proliferation of hPDLCs at the early stage, whereas 8 or 10 μM EGCG remarkably inhibited proliferation. These results are consistent with those of a recent study, in which Jin et al. [27] reported that 5 μM EGCG promoted the cell proliferation of human bone marrow derived mesenchymal cells. Moreover, several studies indicated that EGCG exerted negative effects on the proliferation of osteoblasts in vitro, especially at doses beyond 20 μM [28, 29]. Kamon et al. [20] reported that EGCG had no impact on the proliferation of murine osteoblastic MC3T3-E1 cells. The discrepancies in results between previous studies and the present study may be attributed to the different experimental conditions, such as the cell types and exposure times used.

The influence of EGCG on the proliferation of cells may be due to its effect on intracellular ROS levels, which can cause DNA damage in the nucleus [28–30]. Under normal conditions, cells can eliminate excess ROS to maintain normal biological functions. If not eliminated, superfluous ROS may impair the host DNA, leading to the permanent loss of cell function or even cell death [30]. EGCG has been reported to possess both antioxidant and pro-oxidant effects [16, 31]. At lower concentrations, the antioxidant effect of EGCG was reported to be predominant, leading to its protective effect on DNA. However, EGCG at higher concentrations may possess a relatively stronger ability to exert pro-oxidant action, which may gradually overpower its antioxidant system, resulting in a pro-oxidant effect on DNA. As shown in Fig. 2, compared to the control group, the 2 and 4 μM EGCG treatments had no effects on intracellular ROS generation, and EGCG treatments exceeding 6 μM significantly increased intracellular ROS levels. Simultaneously, after incubation for 72 h with 6 to 10 μM EGCG, the proliferation of hPDLCs was inhibited when compared to that of the control group (Fig. 1A), indicating a cytotoxic effect of EGCG at higher concentrations.

The effects of EGCG on osteogenesis were further evaluated in the present study. ALP activity is commonly used as an indicator of the early stage of osteogenesis because it can provide essential phosphoric acid for the deposition of apatite through hydrolyzing phosphate [32]. As a result of osteogenesis, the formation of mineralizing nodules in the extracellular matrix could be used as an intuitionistic index [33]. Thus, ALP enzyme activity was tested at day 7, which is considered to be an early time point for osteogenesis, and alizarin red S staining was performed after incubation with EGCG for 21 d. The ALP activity assay and alizarin red S staining are considered to be gold standard indicators for the early and late stages of osteogenic differentiation respectively. The results in the present study demonstrated that treatment with 2 to 8 μM EGCG could promote the mineralization of hPDLCs, with 4 to 6 μM EGCG showing the most significant impacts, which is consistent with a previous study using human bone marrow mesenchymal stem cells [27]. However, when using MC3T3-E1 cells, Kamon et al. [20] showed a contradictory result and reported that EGCG at concentrations from 1 to 10 μM suppressed osteogenic differentiation. It appears that the osteogenic responses to EGCG may be dependent on the cell type.

The specific osteogenic genes are expressed at different stages of osteogenic differentiation. During bone development, RUNX2 plays an essential role in the direct initiation of other osteogenic genes, such as ALP and COL1, during the early stage, and OPN and OSX during the late stage [34]. As a recently discovered osteoblast-specific zinc finger-containing transcription factor, OSX is expressed during the late stage of osteogenesis and acts downstream of RUNX2 [35]. COL1, which is the most predominant extracellular protein in bone, initially provides a structural framework for inorganic deposition [32]. Then, the specific binding of OPN to COL1 may naturally orient OPN, influencing osteoblast adhesion, differentiation, and function [36]. In the present study, 4 to 8 μM EGCG up-regulated the mRNA and protein expression levels of most of the tested markers, which supported the results of the ALP activity and mineralization nodule formation studies. The mRNA and protein expression level tendencies were not identical in the present study. This lack of consistency may be caused by different test times or mistakes caused by stochastic post-transcription and post-translation, as well as experimental errors and noise interferences [37].

However, the detailed molecular mechanisms involved in EGCG-induced osteogenic capability in human periodontal ligament cells remain unclear. Previous studies showed that the activation of the Nrf2, Wnt/β-catenin and p44/p42 MAP kinase signaling pathways might be involved in the cellular osteogenic response to EGCG in osteoblasts [38–40]. With this background, EGGC may induce osteogenic differentiation of hPDLCs via the above mechanisms and we plan to elucidate the molecular mechanisms in a future study. Additionally, there are other limitations of the present study. It should be mentioned that most cells in living tissues or organs are capable of reproducing the paracrine and exocrine signals generated by cell-cell and cell-extracellular matrix interactions [41]. These interactions are often critical for guiding and maintaining specific attachment-dependent cellular identities in vivo. Nevertheless, these micro environmental modulators of cell behavior are often missing in dissociated two-dimensional monolayer cell cultures, even in three-dimensional culture models [42]. Thus, further study is needed to clarify the role of EGCG in periodontal bone regeneration in vivo.

## Conclusions

The present study demonstrated that EGCG might promote the osteogenesis of hPDLCs in a dose-dependent manner, suggesting a potential therapeutic role of EGCG as a beneficial supplement when treating patients with periodontal bone loss. The concentration of EGCG should be limited to a certain range in future applications because the concentration affects the osteogenic ability of cells.

## Abbreviations

ALP: Alkaline phosphatase
AO: Acridine orange
CCK-8: Cell Counting Kit-8
COL1: Type I collagen
DCFH-DA: 2′, 7′-Dichlorofluorescein diacetate
EB: Ethidium bromide staining
EGCG: Epigallocatechin-3-gallate
FBS: Fetal bovine serum
GAPDH: Glyceraldehyde-3-phosphate dehydrogenase
hPDLCs: Human periodontal ligament cells
IL-6: Interleukin-6
IQR: Interquartile range
ODM: Osteogenic differentiation medium
OPN: Osteopontin
OSX: Osterix
PVDF: Polyvinylidene difluoride
qRT-PCR: Quantitative real-time polymerase chain reaction
ROS: Reactive oxygen species
RUNX2: Runt-related transcription factor 2
TNF-α: Tumor necrosis factor-α
α-MEM: α-minimum essential medium
